# An Efficient Method for the *N*-Bromosuccinimide Catalyzed Synthesis of Indolyl-Nitroalkanes

**DOI:** 10.3390/molecules14103952

**Published:** 2009-10-09

**Authors:** Chun-Wei Kuo, Chun-Chao Wang, Hu-Lin Fang, B. Rama Raju, Veerababurao Kavala, Pateliya Mujjamil Habib, Ching-Fa Yao

**Affiliations:** Department of Chemistry, National Taiwan Normal University, 88, Sec. 4, Tingchow Road, Taipei 116, Taiwan

**Keywords:** indolyl-nitroalkane, *N*-bromosuccinimide, nitroalkenes, Friedel-Crafts alkylation

## Abstract

An efficient and practical method for the synthesis of indolyl-nitroalkane derivatives catalyzed by *N*-bromosuccinimide is described. The generality of this method was demonstrated by synthesizing an array of diverse 3-substituted indole derivatives by the reaction of different β-nitrostyrenes with various substituted indoles. Simple reaction conditions accompanied by good yields of indolyl-nitroalkanes are the merits of this methodology.

## Introduction

**Figure 1:**
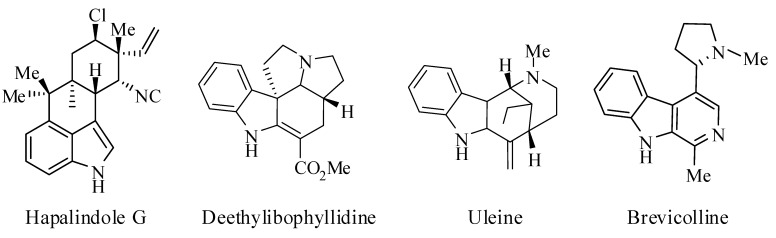
Indole alkaloids.

The indole nucleus is one of the most ubiquitous scaffolds found in natural products, pharmaceuticals, functional materials, and agrochemicals [1,2,3]. Several indole derivatives that occur in nature possess pharmacological activity. These include the hapalindole alkaloids, which exhibit significant antibacterial and antimycotic activity. Other indole alkaloids are uleine, aspidospermidine, the ibophyllidine alkaloids, brevicolline and numerous tryptamine derivatives which exhibit important biological activities (Figure 1) [4,5,6].

Owing to the importance of the indole framework-containing compounds, the development of new strategies to synthesize indole derivatives remain a subject of interest in the present days. During the past several years, Michael addition was the most frequently employed tool for the synthesis of 3-substituted indole derivatives [7]. This is due to the fact that nitroalkenes are very good Michael acceptors and further the Michael adduct of the nitroalkenes are amenable to transformation into a wide range of different functionalized species. Similarly, Friedel-Crafts alkylation of indoles with nitroolefins in the presence of acidic catalysts is well documented in the literature [8,9,10,11,12,13,14,15,16,17,18,19,20,21,22]. Neverthless, most of the procedures reported suffer from several shortcomings such as the use of hazardous catalysts, tedious workup procedures and difficulties in product isolation. The most important drawback is the tendency of electron-rich heteroaromatic rings to undergo polymerization under acid catalyzed conditions. Hence the development of an effective method for the synthesis of 3-substituted indoles has still remained a problem far from resolution. In addition, recent progress in the stereoselective synthesis of these compounds was reported [23,24].

*N*-Bromosuccinimide (NBS), a mild source of bromine, is widely used in the presence of a catalytic amount of free-radical initiators for benzylic and allylic brominations with high regioselectivity [25]. In many instances, NBS have been used as an activator in stereoselective glycosidation [26], protection [27] and deprotection of ketals [28] or THP ethers [29] and in the synthesis of diindolylalkanes [30]. It is also widely employed as a mild oxidant [31] as well as for oxidative cyclizations [32,33]. Recently, we have reported synthesis of 1,5-benzodiazepine derivatives catalyzed by NBS under mild conditions [34]. In continuation of our research work on nitroolefins [35,36,37,38,39,40,41,42,43], we have developed a new route for the synthesis of 3-alkylated indoles via Friedel-Crafts alkylation of indole with β-nitrostyrene, catalyzed by *N*-bromosuccinimide.

## Results and Discussion

**Scheme 1:**
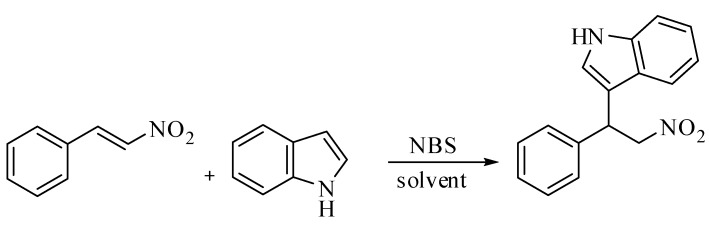
Alkylation of indole with β-nitrostyrene.

Initially, we examined the reaction between β-nitrostyrene and indole with 10 mol% of *N*-bromo-succinimide in dichloromethane at room temperature (Scheme 1). We were able to obtain only 67% of the corresponding alkylated adduct. Further, we examined the fate of the reaction at elevated temperatures. Conducting the reaction at 40 ^o^C afforded the product in 94% yield, after 5 h (entry 3, Table 1). With 20 mol% of *N*-bromosuccinimide, the product yield remained the same, along with the same reaction time as when 10 mol% of NBS was utilized. Our optimization studies showed that 10 mol% of *N*-bromosuccinimide could be the best choice for this transformation, which is evident from Table 1.

**Table 1:** Optimization studies of the alkylation reaction of indole and β-nitrostyrene.

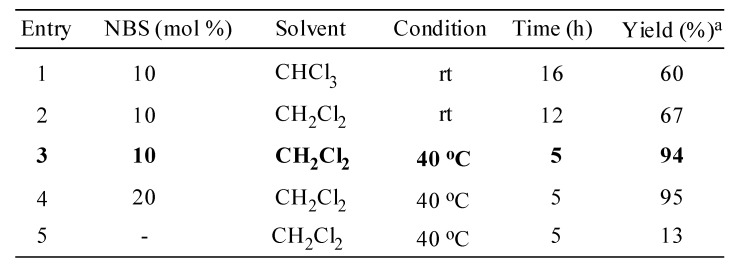

^a^ Yields were determined from ^1^H-NMR with toluene as internal standard.

**Scheme 2:**
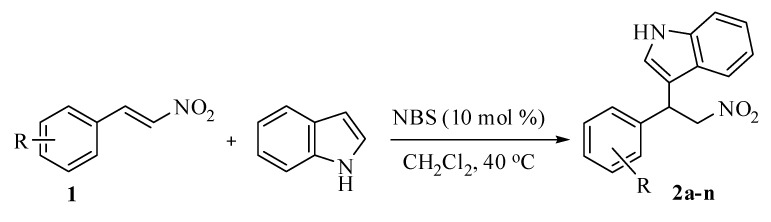
Reaction of indole with various nitroalkenes catalyzed by NBS.

When 1 mmol of β-nitrostyrene **1** was reacted with 1.3 mmol of indole in the presence of 10 mole% of *N*-bromosuccinimide using dichloromethane as solvent at 40 °C, the reaction afforded exclusively the corresponding indolyl-nitroalkane in good to excellent yields. To further explore the scope and limitations of this methodology, we tested the alkylation reaction of indole with a wide array of structurally diverse nitroalkenes (Scheme 2). As seen from Table 2, the alkylation of indoles proceeds with various nitroolefins. The rate of the reaction is controlled by both electronic and steric factors of the substituent attached to the benzene ring of the β-nitrostyrene. For example, the nitroolefins containing electron-withdrawing groups (chloro and nitro, entries 2 and 4, Table 2) react well to afford the 3-alkylated indoles in excellent yields. However, nitroalkenes equipped with strong electron-donating groups such as methyl or methoxy (entries 1 and 3) required relatively longer reaction times. Moreover, sterically hindered substrates (entries 5-8) reacted smoothly under the present reaction conditions. Acid sensitive heterocyclic moieties substrates such as thienyl (entry 9) and furoyl (entry 10) and less reactive aliphatic nitroalkenes (entries 11 and 12) also reacted with equal ease to furnish the Friedel-Crafts products in high yields. 1-Nitrocyclohex-1-ene (entry 13), upon reaction with indole resulted in a mixture of two diastereomers in 7:3 ratio.

**Table 2:** Synthesis of indolyl-nitroalkane derivatives from various nitroalkenes.

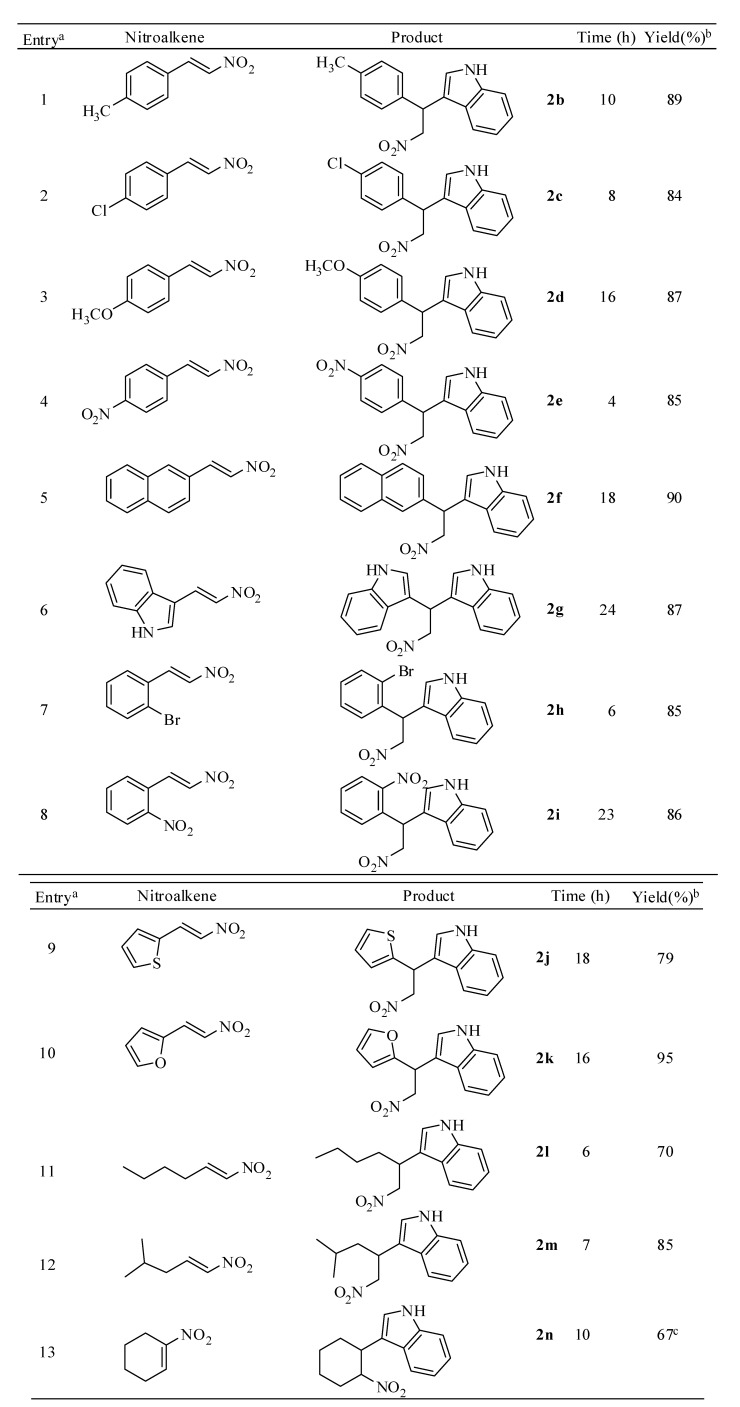

^a^ All reactions were performed by using 1 equiv. of nitroalkene and 1.3 equiv. of indole in the presence of 10 mol% of NBS; ^b^ Isolated yields; ^c^ A mixture of two diastereomers in 7:3 ratio.

**Scheme 3:**
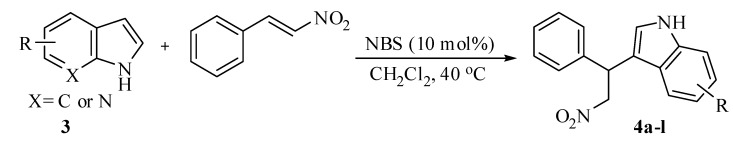
Reaction of β-nitrostyrene with various indoles and other heterocyclic compounds catalyzed by NBS.

Furthermore, to study the effect of the substituents on the heterocyclic substrate, the reaction of β-nitrostyrene with various substituted indole and other heterocyclic derivatives was examined (Scheme 3). 2-Methylindole was reacted with β-nitrostyrene to furnish the product **4b** in high yield. Similarly, 5-methoxyindole bearing an electron-donating group (entry 5, Table 3) was alkylated with β-nitrostyrene in a shorter time to give the desired product **4e** in excellent yield. Moreover, increasing the size of the substituent on the indole nucleus, such as in the case of 2-phenylindole, gave only 47% yield. Generally, alkylation of hydroxyindoles with nitroalkenes often results in various side products, thereby decreasing the yields of alkylated product which may be due to the interaction of the hydroxyl group with the Lewis acid catalyst [44]. Indole containing free or protected hydroxyl group (entries 6, 8 and 9) reacted with the nitroolefin without any interference of the functional group. It is interesting to note that fused heteroarenes such as 7-azaindole (entry 10, Table 3) reacted with β-nitrostyrene to afford the corresponding adduct in moderate yield after prolonged reaction time. This method was found to be quite successful with the highly nucleophilic substrate pyrrole (entry 11) and the acid sensitive substrate 2-methoxyfuran (entry 12), producing the expected products in better yields compared to the earlier reported methods [45].

**Table 3:** Synthesis of aryl-nitroalkane derivatives from various indoles and other heterocyclic derivatives.

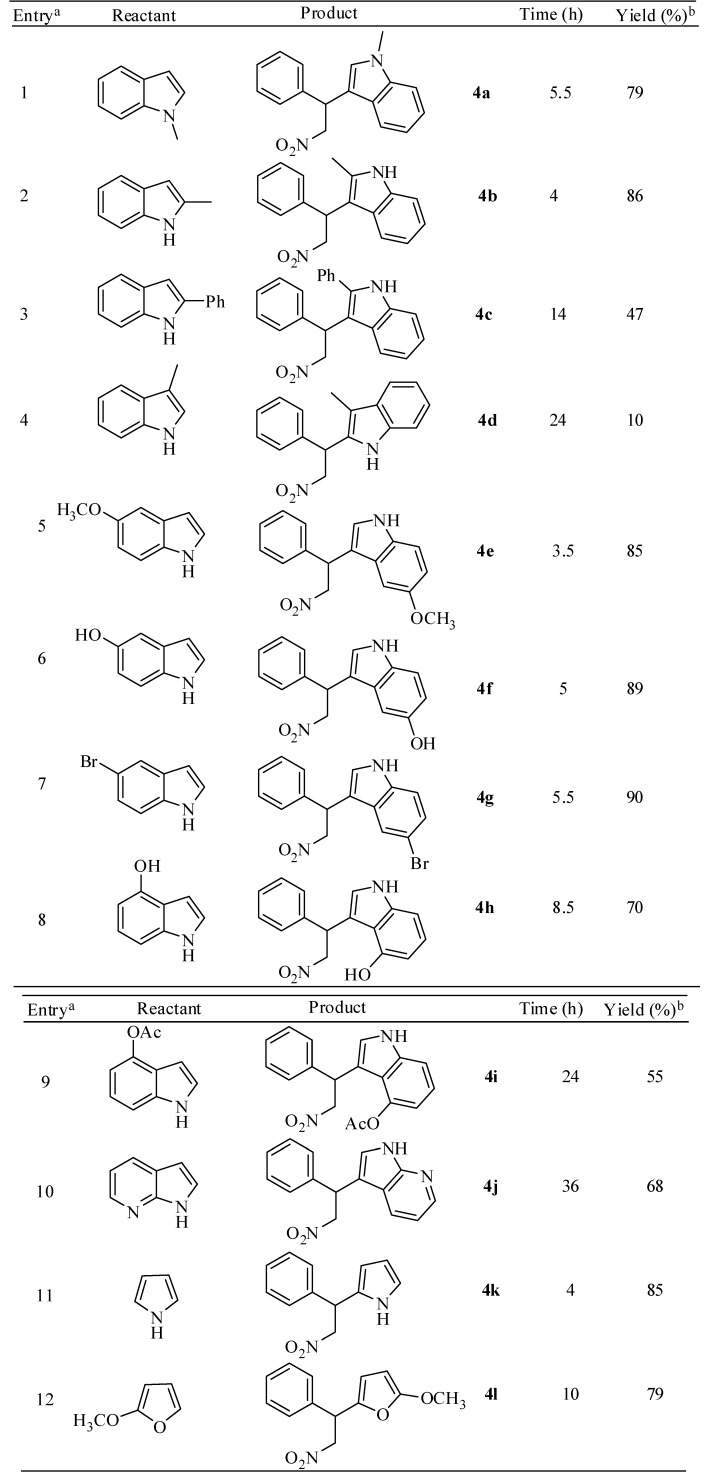

^a^ All reactions were carried out by using 1 equiv. of nitroalkene and 1.3 equiv. of indole in the presence of 10 mol% of NBS; ^b^ isolated yields.

**Scheme 4:**
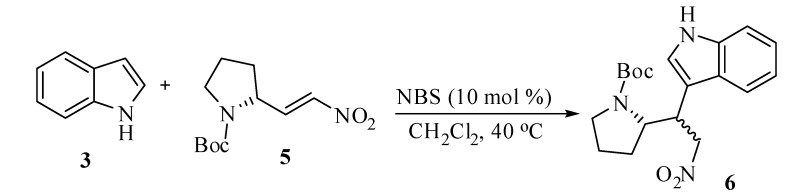
Synthesis of the key precursor of (*S*)-brevicolline.

An interesting application of this methodology involves the synthesis of the compound **6**, which is the key precursor for the synthesis of the natural product (*S*)-brevicolline. The nitroalkene **5** was prepared by the condensation of Boc-*S*-pyrrolidine-2-carboxyaldehyde with nitromethane according to the reported procedure [46]. Compound **6** was obtained as a mixture of two diastereomers in the ratio of 9:1 (HPLC) in 80% yield by the reaction of indole with nitroalkene **5** under the present reaction conditions in 16 h. The aforementioned example demonstrates the usefulness and versatility of this synthetic methodology.

## Conclusion

In conclusion, we have developed a simple and efficient method for the synthesis of indolyl-nitroalkanes in good yields under catalytic conditions. This method is applicable to a wide range of nitroalkenes and various indoles. Low cost of the reagents and convenient isolation process of this reaction makes this method an attractive alternative to existing methods.

## Experimental

### General

All reagents and chemicals were purchased from Sigma-Aldrich Chemical Company, Acros Organics, Alfa Aesar or Merck and were used as received. Analytical thin layer chromatography was performed with E. Merck silica gel 60F glass plates and flash chromatography with E. Merck silica gel 60 (230-400 mesh). Melting points were determined with a microscope hot-stage apparatus and uncorrected. ^1^H- and ^13^C-NMR spectra were recorded on a Bruker Avance 400 instrument. Chloroform-*d* was used as the solvent and TMS (δ = 0.00 ppm) as an internal standard. Chemical shift values are reported in ppm relative to TMS in delta (δ) units. Chemical shifts are recorded in parts per million (ppm). For ^1^H-NMR spectra the residual solvent peak was used as an internal reference (CHCl_3_ 7.26). For ^13^C-NMR spectra the central peak of the CDCl_3_ triplet was used as the internal reference (77.26). Multiplicities are recorded as s, singlet; d, doublet; t, triplet; q, quartet; dd, doublet of doublet; br broadened; m, multiplet. Coupling constants (J) are expressed in Hertz. MS and HRMS were measured on JEOL JMS-D300 and JEOL JMS-HX110 spectrometers, respectively.

### General procedure for the synthesis of aryl-nitroalkanes

To a stirred solution of nitroalkene derivative (1 mmol) and indole (1.3 mmol) in CH_2_Cl_2_ (2 mL) was added NBS (0.1 mmol). The reaction mixture was then warmed to 40 °C with continuous stirring until the completion of the reaction as monitored by TLC. Upon completion, the reaction mixture was diluted with water and extracted with CH_2_Cl_2_ (3 x 15 mL), washed with brine, and then dried over MgSO_4_. The solvent was removed under reduced pressure and the crude residue was purified by flash chromatography on silica gel to afford pure product. The references of the known compounds are: **2a** [14,15,17,19], **2b** [36], **2c** [15,22], **2d** [13,15,17], **2e** [36], **2f** [36], **2g** [22], **2j** [15,22], **2k** [15,36], ***cis-*2n** [14], ***trans-*2n** [14], **4a** [15,17,19], **4b** [15,17], **4c** [12,36], **4d** [15], **4e** [14,17], **4f** [14], **4g** [17,19], **4k** [15,35], **4l** [45], **5** [46].

*3-(1-(2-Bromophenyl)-2-nitroethyl)-1H-indole* (**2h**): Colorless solid. mp 117-118 °C. ^1^H-NMR (400 MHz, CDCl_3_): δ 8.07 (brs, 1H), 7.61 (d, *J* = 7.8 Hz, 1H), 7.42 (d, *J* = 8.0 Hz, 1H), 7.33 (d, *J* = 8.2 Hz, 1H), 7.20-7.18 (m, 3H), 7.12-7.05 (m, 3H), 5.72 (t, *J* = 8.0 Hz, 2H), 4.99-4.90 (m, 1H); ^13^C NMR (100 MHz, CDCl_3_): δ 138.3 (C), 136.7 (C), 133.7 (C), 129.4(CH), 129.3 (CH), 128.1 (CH), 126.4 (CH), 124.7 (CH), 123.0 (CH), 122.1 (C), 120.2 (CH), 119.2 (CH), 113.6 (C), 111.6 (CH), 78.0 (CH_2_), 40.8 (CH); MS (EI) *m/z* (relative intensity): 346 (14) [M+2]^+^, 344 (13) [M]^+^, 297 (28), 218 (100), 204 (27), 108 (20); HRMS-EI: *m/z* [M]^+^ calcd. for C_16_H_13_BrN_2_O_2_: 344.0160; found: 344.0155.

*3-(2-Nitro-1-(2-nitrophenyl)ethyl)-1H-indole* (**2i**): Yellow solid. mp 117-119 °C. ^1^H-NMR (400 MHz, CDCl_3_): δ 8.20 (brs,1H), 7.89 (d, *J* = 7.8 Hz, 1H), 7.48-7.37 (m, 3H), 7.32 (dd, *J* = 12.7 and 8.4 Hz, 2H), 7.18 (t, *J* = 7.6 Hz, 1H), 7.13 (s, 1H), 7.03 (t, *J* = 7.3 Hz, 1H), 5.87 (t, *J* = 7.7 Hz, 1H), 5.09 (m, 2H). ^13^C-NMR (100 MHz, CDCl_3_): δ 149.8 (C), 136.7 (C), 133.9 (C), 133.4 (C), 130.1 (CH), 128.8 (CH), 126.1 (CH), 125.3 (CH), 123.2 (CH), 122.3 (CH), 120.4 (CH), 118.8 (CH), 113.0 (C), 111.7 (CH), 78.3 (CH_2_), 36.6 (CH); MS (EI) *m/z* (relative intensity): 311 (19) [M]^+^, 264 (29), 247 (33), 219 (100), 204 (39), 130 (57); HRMS-EI: *m/z* [M]^+^ calcd for C_16_H_13_N_3_O_4_: 311.0906; found: 311.0901.

*3-(1-Nitromethylpentyl)-1H-indole* (**2l**): ^1^H-NMR (400 MHz, CDCl_3_): δ 8.06 (brs, 1H), 7.61 (d, *J* = 7.8 Hz, 1H), 7.34 (d, *J* = 8.1 Hz, 1H), 7.23-7.18 (m, 1H), 7.15-7.11 (m, 1H), 6.98 (d, *J* = 2.0 Hz, 1H), 4.59 (m, 2H), 3.86 (m, 1H), 1.89 (m, 1H), 1.52 (m, 2H), 0.89 (d, *J* = 6.0 Hz, 3H), 0.87 (d, *J* = 6.0 Hz, 3H); ^13^C-NMR (100 MHz, CDCl_3_): δ 136.5 (C), 126.1 (C), 122.4 (CH), 122.1 (CH), 119.7 (CH), 118.7 (CH), 114.1 (C), 111.5 (CH), 81.0 (CH_2_), 41.4 (CH), 34.4 (CH_2_), 25.4 (CH_2_), 23.3 (CH_2_), 21.5 (CH_3_); MS (EI) *m/z* (relative intensity): 246 (19) [M]^+^, 178 (11), 157 (30), 144 (18), 143 (61), 130 (100), 115 (22), 77 (10); HRMS-EI: *m/z* [M]^+^ calcd for C_14_H_18_O_2_N_2_: 246.1368; found: 246.1370.

*3-(3-Methyl-1-nitromethylbutyl)-1H-indole* (**2m**): ^1^H-NMR (400 MHz, CDCl_3_): δ 8.07 (brs, 1H), 7.62 (d, *J* = 7.8 Hz, 1H), 7.33 (d, *J* = 8.1 Hz, 1H), 7.20 (m, 1H), 7.13 (m, 1H), 6.99 (d, *J* = 2.2 Hz, 1H), 4.59 (m, 2H), 3.87 (m, 1H), 1.89 (m, 1H), 1.51 (m, 2H), 0.88 (dd, *J* = 6.0 and 5.7 Hz, 6H); ^13^C-NMR (100 MHz, CDCl_3_): δ 136.5 (C), 126.1 (C), 122.3 (CH), 119.7 (CH), 118.7 (CH), 113.9 (C), 111.5 (CH), 80.9 (CH_2_), 41.4 (CH_2_), 34.4 (CH), 25.4 (CH), 23.3 (CH), 21.5 (CH_3_); MS (EI) *m/z* (relative intensity): 246 (24) [M]^+^, 157 (26), 144 (36), 143 (71), 130 (100), 115 (24), 84 (19), 57 (31), 55 (25); HRMS-EI: *m/z* [M]^+^ calcd for C_14_H_18_O_2_N_2_: 246.1368; found: 246.1365.

*3-(2-Nitro-1-phenylethyl)-1H-indol-4-ol* (**4h**): ^1^H-NMR (400 MHz, CDCl_3_): δ 7.94 (brs, 1H), 7.31-7.24 (m, 4H), 7.18 (d, *J* = 9.4 Hz, 1H), 6.93 (t, *J* = 7.8 Hz, 1H), 6.85 (d, *J* = 8.1 Hz, 1H), 6.64 (s, 1H), 6.32 (d, *J* = 7.5 Hz, 1H), 5.43 (t, *J* = 8.1 Hz, 1H), 5.23 (dd, *J* = 12.7 and 6.6 Hz, 1H), 5.11 (brs, 1H), 4.87 (t, *J* = 11.8 Hz, 1H); ^13^C-NMR (100 MHz, CDCl_3_): δ 149.7 (C), 139.7 (C), 138.9 (C), 128.8 (CH), 127.8 (CH), 127.4 (CH), 123.4 (CH), 121.8 (C), 115.5 (CH), 114.7 (C), 104.8 (CH), 104.5 (CH), 79.8 (CH_2_), 42.1 (CH); MS (EI) *m/z* (relative intensity): 282 (54) [M]^+^, 236 (47), 235 (100), 234 (32), 222 (70), 220 (45), 117 (14), 103 (17), 77 (12); HRMS-EI: *m/z* [M]^+^ calcd for C_16_H_14_O_3_N_2_: 282.1004; found: 282.1000.

*3-(2-Nitro-1-phenylethyl)-1H-indol-4-yl acetate* (**4i**): ^1^H-NMR (400 MHz, CDCl_3_): δ 8.16 (brs, 1H), 7.27-7.06 (m, 7H), 6.79 (d, *J* = 6.8 Hz, 1H), 6.65 (s, 1H), 5.19 (m, 1H), 5.01 (dd, *J* = 12.8 and 6.5 Hz, 1H), 4.85 (dd, *J* = 11.8 and 9.9 Hz, 1H), 2.21 (s, 3H); ^13^C-NMR (100 MHz, CDCl_3_): δ 170.3 (C), 143.8 (C), 139.0 (C), 138.9 (C), 129.0 (CH), 127.8 (CH), 127.7 (CH), 124.0 (CH), 122.8 (CH), 118.5 (CH), 113.2 (C), 112.6 (CH), 109.7 (CH), 79.9 (CH_2_), 42.0 (CH), 21.3 (CH_3_); MS (EI) *m/z* (relative intensity): 324 (16) [M]^+^ , 282 (68), 236 (55), 235 (100), 222 (49); HRMS-EI: *m/z* [M]^+^ calcd for C_18_H_16_N_2_O_4_: 324.1110; found: 324.1111.

*3-(2-Nitro-1-phenylethyl)-1H-pyrrolo[2,3-b]pyridine* (**4j**): ^1^H-NMR (400 MHz, CDCl_3_): δ 8.31 (d, *J* = 3.5 Hz, 1H), 7.87 (dd, *J* = 7.7 and 1.1 Hz, 1H), 7.33-7.30 (m, 5H), 7.20 (d, *J* = 3.5 Hz, 1H), 7.07 (dd, *J* = 7.7 and 4.7 Hz, 1H), 6.71 (dd, *J* = 9.2 and 6.1 Hz, 1H), 6.47 (d, *J* = 3.6 Hz, 1H), 5.37 (dd, *J* = 13.4 and 9.2 Hz, 1H), 5.16 (dd, *J* = 13.4 and 6.1 Hz, 1H). ^13^C-NMR (100 MHz, CDCl_3_): δ 147.6 (C), 143.2 (CH), 135.9 (C), 129.3 (CH), 129.1 (CH), 129.0 (CH), 128.8 (CH), 127.0 (CH), 126.0 (CH), 121.0 (C), 116.6 (CH), 101.4 (C), 56.7 (CH_2_); MS (EI) *m/z* (relative intensity): 267 (5) [M]^+^, 221 (21), 149 (11), 118 (100), 102 (18), 91 (70), 84 (25), 77 (45), 63 (21), 51 (28); HRMS-EI: *m/z* [M]^+^ calcd for C_15_H_13_O_2_N_3_: 267.1008; found: 267.1005.
